# Solvent demulsification-dispersive liquid-liquid microextraction based on solidification of floating organic drop coupled with ultra-high-performance liquid chromatography-tandem mass spectrometry for simultaneous determination of 13 organophosphate esters in aqueous samples

**DOI:** 10.1038/s41598-019-47828-8

**Published:** 2019-08-05

**Authors:** Qing Luo, Shiyu Wang, Muhammad Adeel, Yue Shan, Hui Wang, Li-na Sun

**Affiliations:** 1 Key Laboratory of Regional Environment and Eco-Remediation of Ministry of Education, College of Environment, Shenyang University, Shenyang, 110044 China; 2 Beijing Key Laboratory of Farmland Soil Pollution Prevention and Remediation, College of Resources and Environmental Sciences, China Agricultural University, Beijing, 100193 China

**Keywords:** Mass spectrometry, Environmental monitoring

## Abstract

This study developed a novel method for the determination of 13 organophosphate esters (OPEs) in aqueous samples through the optimization of solvent demulsification-dispersive liquid-liquid microextraction based on solidification of floating organic drop procedure coupled with ultra-high-performance liquid chromatography-tandem mass spectrometry. The proposed method was rapid and accurate and could be used in field applications. Under the most suitable conditions, the limit of detection and limit of quantification ranged from 0.16 ng/L to 20.0 ng/L and from 0.55 ng/L to 66.7 ng/L, respectively. The enrichment factors (EFs) ranged from 30 to 46. The relative standard deviations were less than 15%. The spiked recoveries ranged between 68.2% and 97.7% in the analysis of actual aqueous samples. The proposed method was convenient, environment friendly, and time and solvent saving and could be used in field applications compared with other methods. Various concentrations and types of OPEs were detected in tap water, river water, and effluent of sewage treatment plant. Effluent samples had the highest detected levels and types of OPEs.

## Introduction

Organophosphate esters (OPEs) are extensively applied in textile, electronics, plastics, and other industries as flame retardants or plasticizers^1^. Generally, OPEs are added to the final product rather than chemically bonded. This process allows OPEs to be easily released to the surrounding environment^2^. Recently, OPEs have been detected in different environmental media, such as wastewater^3^, sludge^4^, surface water^5–7^, sediment^8–10^, soil^11–13^, indoor and outdoor air, airborne particulate matter, and dust^14–21^. OPEs have potential adverse effects on the ecosystem and human health. For instance, chlorinated OPEs, such as tricresyl phosphate (TCrP) and tributyl phosphate (TnBP), are potential human carcinogens and may have reproductive toxicity^22,23^.

Generally, the concentrations of OPEs are low in environmental media; thus, an efficient pretreatment approach is usually required for the determination of OPEs. Different pretreatment techniques, including liquid-liquid extraction (LLE), solid-phase extraction (SPE), solid-phase microextraction (SPME), dispersive liquid–liquid microextraction (DLLME), and DLLME based on solidification of floating organic drop (DLLME-SFO), have been applied for the evaluation of OPEs in aqueous samples^24–32^. However, these methods have different disadvantages. For example, LLE usually requires a large volume of samples and toxic organic solvents. SPE also requires a large volume of samples and is prone to clogging. SPME usually consumes considerable time, and the fibers are fragile. DLLME is an effective method that only requires small amounts of samples and solvents. However, it frequently uses halohydrocarbons as the extractant, which are highly toxic and environmentally harmful. DLLME-SFO is a modified DLLME that uses solvents with low density, proper melting point, and low toxicity as the extractant. However, it requires centrifugation to separate organic and aqueous phases. Solvent demulsification–DLLME-SFO (SD-DLLME-SFO) is an improved method that can avoid these drawbacks. It uses demulsifiers rather than centrifugation to separate organic and aqueous phases, which makes it suitable in field analysis^33^. SD-DLLME-SFO has been successfully used in the analysis of organic compounds, including organochlorine pesticides, polycyclic aromatic hydrocarbons, and sulfonylurea herbicides^34–36^. However, it has not been applied for the determination of OPEs.

In this study, SD-DLLME-SFO coupled with ultra-high-performance liquid chromatography–tandem mass spectrometry (UHPLC-MS/MS) was developed for the determination of OPEs in aqueous samples. Three water samples collected from different sources were selected to evaluate the efficiency of the proposed method and analyze the concentrations of OPEs in real samples.

## Results and Discussion

### Optimization of SD-DLLME-SFO conditions

Variables affecting the extraction performance, including the type and volume of extractant, dispersant, and demulsifier; extraction time; and pH of samples, were investigated to obtain the suitable extraction efficiency. Ionic strength influences the extraction efficiency, and salt deposited on the transfer line affects the analysis results of UHPLC-MS/MS. Suitable extraction temperature can enhance the extraction efficiency. However, the extraction temperature is difficult to accurately control in DLLME because of the short extraction time. Thus, ionic strength and extraction temperature were not investigated in this study.

**Figure 1:**
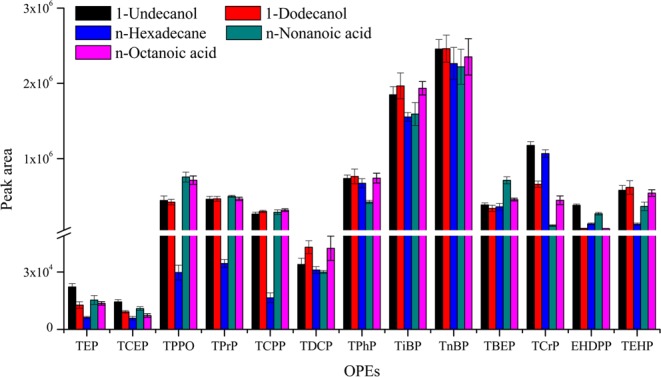
The efficiency of different extraction solvents on the extraction of OPEs from aqueous samples (n = 3).

In SD-DLLME-SFO, the extraction solvent is the primary factor that affects extraction efficiency. It has lower density than water, melting point near room temperature, low solubility in water, and good extraction capacity for the analytes. In this study, the extraction efficiencies of 1-undecanol, 1-dodecanol, n-hexadecane, n-nonanoic acid, and n-octanoic acid were compared. The results are shown in Fig. 1. The difference in extraction efficiency of five extraction solvents was small for most OPEs. The extraction efficiencies of n-hexadecane for triphenylphosphine oxide (TPPO), tripropyl phosphate (TPrP), and tris-(1-chloro-2-propyl)phosphate (TCPP) were significantly lower compared with those of the other four extraction solvents. The extraction efficiencies of 1-undecanol for triethyl phosphate (TEP) and tris-(2-chloroethyl)phosphate (TCEP) were better than those of the other four extraction solvents, which was probably because TEP and TCEP can effectively transfer from water to 1-undecanol. Thus, 1-undecanol was used as the extractant to extract OPEs in aqueous samples.

**Figure 2:**
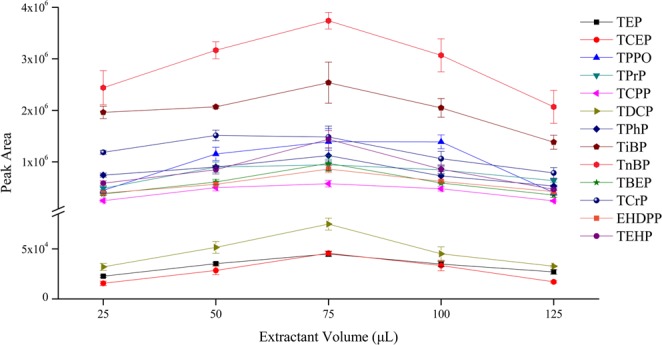
The efficiency of different extractant volumes on the extraction of OPEs from aqueous samples (n = 3).

The volume of extractant should be determined to achieve the best extraction efficiency. Few extractants cannot efficiently extract the analytes, whereas a considerable amount of extractants can increase the extraction amount but impair the enrichment of analytes. In addition, the appropriate volume ratio of extractant and disperser is conducive to form a fine cloudy dispersion of microdroplets. In this study, the volume of 1-undecanol was investigated in the range of 25–125 μL, as presented in Fig. 2. As shown in the figure, the extraction efficiency increased with the increase of extraction volume. The best extraction efficiency was obtained when the extraction volume increased to 75 μL. However, when the extraction volume was further increased, the extraction efficiency decreased. The reason may be because considerable amounts of extraction solvents extracted many analytes but lowered their concentration in the final extract. Thus, 75 μL of 1-undecanol was adopted in this study.

**Figure 3:**
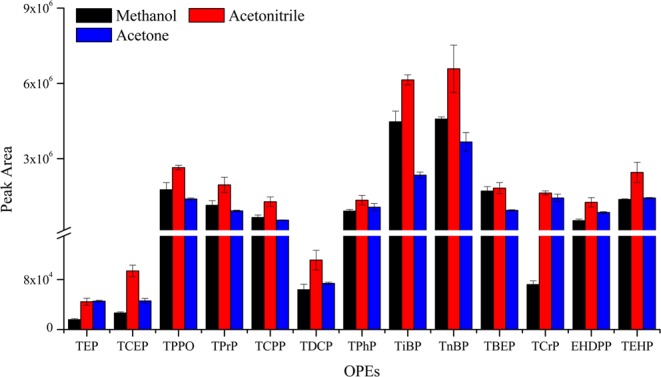
The efficiency of different dispersive solvents on the extraction of OPEs from aqueous samples (n = 3).

In SD-DLLME-SFO, the dispersive solvent should have good solubility in the extractant and water phase to promote the formation of microdroplets of the extractant. In this study, methanol, acetonitrile, and acetone were evaluated, as presented in Fig. 3. The difference in extraction efficiency of the three dispersive solvents was small for most OPEs. The extraction efficiencies of methanol for TEP and TPrP were significantly lower compared with those of acetonitrile and acetone. However, acetonitrile showed better results for TCEP and tris[2-chloro-1-(chloromethyl) ethyl]phosphate (TDCP) compared with methanol and acetone. The reason may be because acetonitrile can promote the dispersion of 1-undecanol in water, increase the contact between 1-undecanol and OPEs, and facilitate the transfer of OPEs from aqueous phase to organic phase. Therefore, acetonitrile was used as the dispersive solvent in this study.

**Figure 4:**
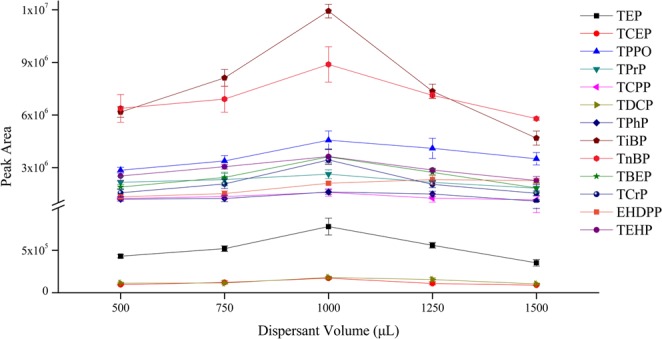
The efficiency of different dispersant volumes on the extraction of OPEs from aqueous samples (n = 3).

The volume of dispersive solvent has a major impact on SD-DLLME-SFO. Low amount of dispersive solvent hardly forms microdroplets of the extractant, whereas excess amount of dispersive solvent increases extractant solubility in aqueous samples, reducing the extraction efficiency. In this study, the volume of acetonitrile was investigated in the range of 500–1500 μL. Figure 4 shows that the extraction efficiency increased with the increase of dispersant volume. The best extraction efficiency was obtained when the dispersant volume increased to 1000 μL. However, when the extraction volume further increased, the extraction efficiency decreased. The reason may be that an appropriate amount of dispersant can promote the dispersion of extractants and form many microdroplets, increasing the extraction efficiency. However, excessive amount of dispersant can lead to the dissolution of extractant in aqueous samples, decreasing the extraction efficiency. Thus, the dispersant volume should be appropriate, and 1000 μL of acetonitrile was adopted in this study.

**Figure 5:**
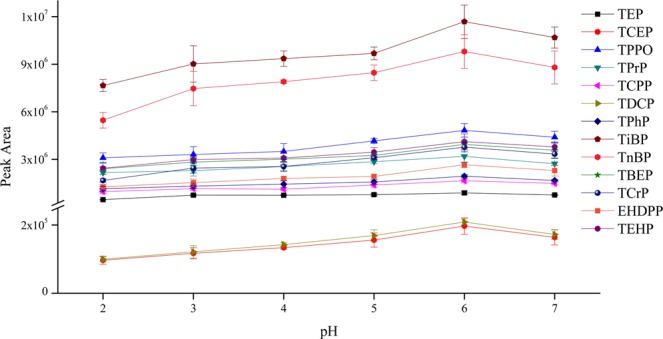
The efficiency of different pH on the extraction of OPEs from aqueous samples (n = 3).

The pH of samples can also influence the solubility of analytes in aqueous samples. Most triesters are stable in neutral and acidic media but can hydrolyze in alkaline media^22,37^. Thus, the pH value was optimized in the range of 2–7 in this study. As shown in Fig. 5, the extraction efficiency for most OPEs had a distinct enhancement when the pH of aqueous samples was adjusted from 2 to 3. However, when the pH was 3, 4, or 5, the difference in extraction efficiency was small. The extraction efficiency improved when the pH was adjusted to 6. However, when the pH increased to 7, the extraction efficiency decreased. The reason may be that under weak acid condition, the solubility of OPEs in organic solvent increased. Thus, the pH of aqueous samples was adjusted to 6 in this study.

**Figure 6:**
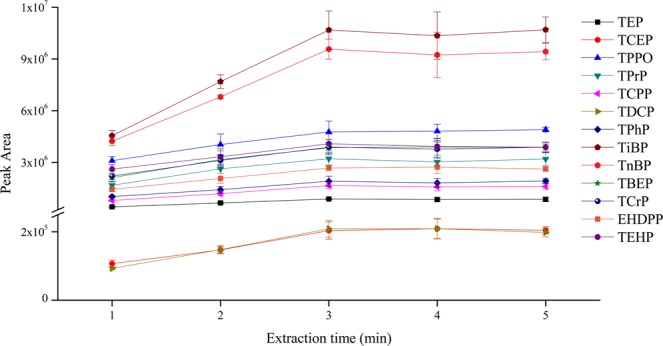
The efficiency of different extraction time on the extraction of OPEs from aqueous samples (n = 3).

SD-DLLME-SFO is a time-saving sample pretreatment method. The extraction equilibrium of analytes in aqueous samples and extractant can be rapidly obtained. In this study, the extraction time was investigated in the range of 1–5 min. As shown in Fig. 6, the extraction efficiency had a distinct enhancement when the extraction time increased from 1 min to 3 min. However, when the extraction time was further increased, the extraction efficiency showed slight changes. This condition indicated that the extraction achieved equilibrium when the extraction time was 3 min. Thus, the extraction time was set to 3 min in this study.

**Figure 7:**
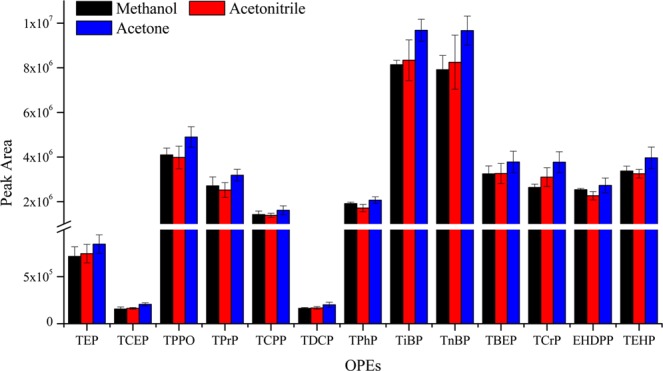
The efficiency of different demulsifiers on the extraction of OPEs from aqueous samples (n = 3).

In SD-DLLME-SFO, demulsifiers were used to break the emulsification system, which accelerated the separation of organic and aqueous phases. In this study, methanol, acetonitrile, and acetone were utilized. Figure 7 shows that the difference in extraction efficiency of the three demulsifiers was very low. The extraction efficiency of acetone was relatively better than those of others. The reason may be because methanol and acetonitrile increased the solubility of OPEs in water. Thus, acetone was used as demulsifier in this study.

**Figure 8:**
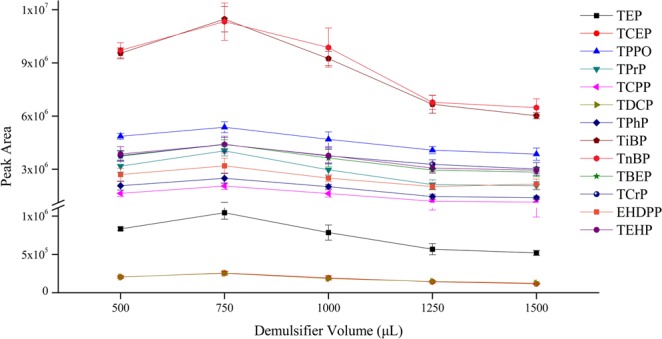
The efficiency of different demulsifier volumes on the extraction of OPEs from aqueous samples (n = 3).

The volume of demulsifier also has a significant impact on SD-DLLME-SFO. On the one hand, the demulsification effect is poor when the demulsifier dosage is insufficient. This condition leads to the low recovery of extractant and decreases the extraction efficiency. On the other hand, excessive demulsifier dosage has dispersive solvent effect, increases the solubility of analytes in aqueous phase, and reduces the extraction efficiency. In this study, the volume of acetone was investigated in the range of 500–1500 μL. The extraction efficiency had a distinct enhancement when the demulsifier volume increased from 500 μL to 750 μL. The extraction efficiency was the highest at 750 μL. However, when the demulsifier volume was further increased, the extraction efficiency decreased (Fig. 8). The reason may be that excessive demulsifier volume can increase the solubility of extractant and analyte in the aqueous phase. In particular, when the demulsifier volume was 1500 μL, the demulsification effect was poor, leading to small amounts of extractant collected. Thus, 750 μL of acetone was adopted in this study.

### Method evaluation

**Figure 9:**
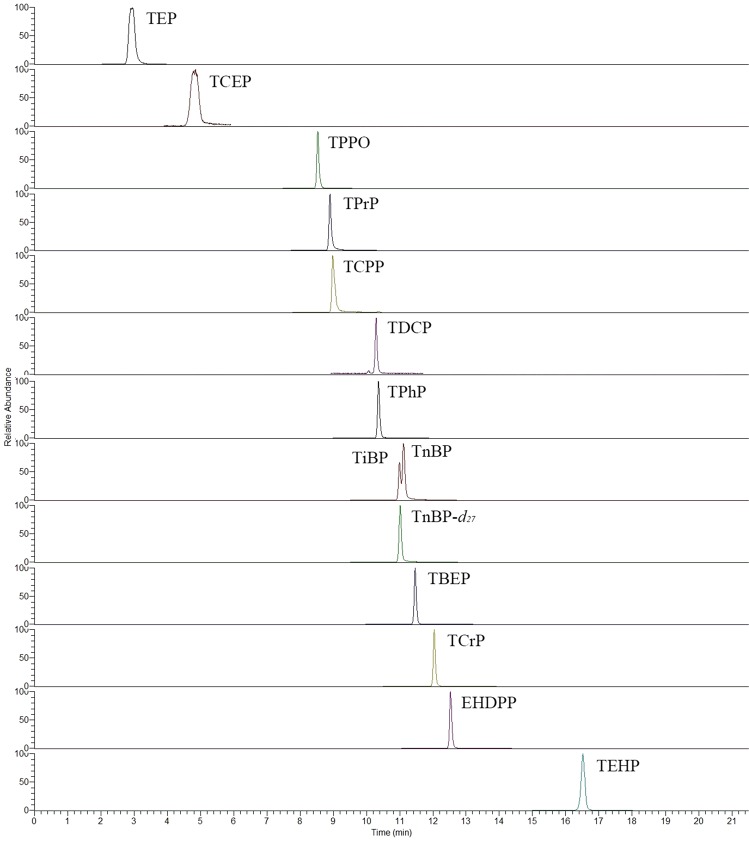
UHPLC-MS/MS chromatograms of 13 OPEs obtained for the spiked aqueous samples (1 µg/L) using the SD-DLLME-SFO method.

A series of aqueous samples containing various concentrations of analytes was prepared and extracted three times for each concentration under the most suitable experimental conditions. The working curves were made and are shown in Table 1. The results showed that the linearity of analytes was better in certain concentration range, and the correlation coefficient (*R*) ranged from 0.9901 to 0.9998. By constantly diluting the concentration of analytes in aqueous samples, the limit of detection (LOD, signal-to-noise ratio (S/N) = 3) and limit of quantification (LOQ, S/N = 10) were 0.16–20.0 and 0.55–66.7 ng/L, respectively. The precision (relative standard deviation, RSD) and enrichment factor (EF) were determined by performing seven repetitions for spiked aqueous samples (1 μg/L). The intraday and interday RSDs were less than 15%. The EFs ranged from 30 to 46. The UHPLC-MS/MS chromatogram of 13 OPEs obtained for the spiked aqueous samples (1 μg/L) is shown in Fig. 9.

**Table 1:** The linear range, correlation coefficient, MDL, MQL, recovery and repeatability for 13 OPEs.

Compounds	Linear range (µg/L)	*R*	MDL (ng/L)	MQL (ng/L)	Intra-day precision (RSD%, n = 7)	Inter-day precision (RSD%, n = 7)	Enrichment Factor (mean ± SD, n = 7)
TEP	0.01–10	0.9937	1.76	5.88	8.09	12.6	37 ± 3
TCEP	0.1–100	0.9901	19.8	65.9	7.77	14.8	31 ± 2
TPPO	0.01–10	0.9966	0.35	1.17	5.74	8.96	46 ± 3
TPrP	0.01–10	0.9969	0.55	1.84	6.93	11.1	37 ± 3
TCPP	0.01–10	0.9961	3.26	10.9	9.71	12.7	39 ± 4
TDCP	0.1–100	0.9987	20.0	66.7	12.2	4.81	30 ± 4
TPhP	0.01–10	0.9998	1.14	3.79	11.9	6.35	38 ± 5
TiBP	0.01–10	0.9952	0.18	0.61	6.29	4.32	40 ± 3
TnBP	0.01–10	0.9958	0.16	0.55	9.32	11.2	36 ± 3
TBEP	0.01–10	0.9941	0.64	2.13	5.64	13.6	35 ± 2
TCrP	0.01–10	0.9922	0.96	3.19	8.92	10.5	33 ± 3
EHDPP	0.01–10	0.9919	0.61	2.03	13.7	8.02	39 ± 5
TEHP	0.01–10	0.9965	0.90	3.00	10.8	12.2	42 ± 5

### Analysis of real samples

To verify the accuracy and practicability of the proposed method, three different aqueous samples (tap water, river water, and effluent of sewage treatment plant) were selected for SD-DLLME-SFO coupled with UHPLC-MS/MS analysis. The effluent of sewage treatment plant, which has a complex matrix, was used to evaluate the matrix effect of the proposed method. The results are shown in Table 2. The matrix effects ranged from 84.7% to 97.9%, indicating that the matrix effect was acceptable in this study. The spiked recoveries (two different spiked concentrations) from tap water, river water, and effluent of sewage treatment plant were 68.2–95.2%, 76.8–93.9%, and 68.5–97.7%, respectively. Their RSDs were less than 15%.

**Table 2:** The concentrations and recoveries of 13 OPEs in three different aqueous samples. ^a^nd = not detected.

OPEs	Spiked (ng/L)	Tap water (n = 3)		RSD (%)	River water (n = 3)			Effluent (n = 3)			Matrix effect (n = 3)	
		Found (ng/L)	Recovery (%)		Found (ng/L)	Recovery (%)	RSD (%)	Found (ng/L)	Recovery (%)	RSD (%)	5 ng (%)	20 ng (%)
TEP	0	24.2 ± 1.97	—	8.13	36.7 ± 4.47	—	12.2	62.3 ± 5.03	—	8.07	85.1 ± 4.81	84.7 ± 3.94
	50	58.3 ± 4.86	68.2	8.33	75.4 ± 7.18	77.3	9.53	99.4 ± 7.81	74.2	7.86		
	200	175.1 ± 11.5	75.5	6.56	197.9 ± 11.3	80.6	5.70	199.2 ± 18.0	68.5	9.02		
TCEP	0	< LOQ	—	—	< LOQ	—	—	125.6 ± 10.8	—	8.57	92.7 ± 5.46	91.1 ± 5.98
	500	419.4 ± 34.6	83.9	8.26	458.0 ± 55.2	91.6	12.1	523.3 ± 25.5	79.5	4.87		
	2000	1746.3 ± 173.3	87.3	9.92	1693.0 ± 151.2	84.7	8.93	1733.5 ± 170.8	80.4	9.85		
TPPO	0	nd^a^	—	—	45.6 ± 4.17	—	9.15	84.5 ± 8.02	—	9.49	93.8 ± 6.84	92.0 ± 3.71
	50	44.1 ± 4.20	88.2	9.52	89.4 ± 10.0	87.7	11.2	124.9 ± 8.72	80.8	6.98		
	200	181.7 ± 10.6	90.9	5.85	233.3 ± 30.1	93.8	12.9	264.3 ± 19.3	89.9	7.30		
TPrP	0	< LOQ	—	—	< LOQ	—	—	18.6 ± 0.42	—	2.27	86.2 ± 5.71	87.0 ± 3.89
	50	43.5 ± 2.55	86.9	5.87	43.2 ± 2.04	86.3	4.72	63.5 ± 8.26	89.8	13.0		
	200	183.4 ± 9.37	91.7	5.11	181.2 ± 20.9	90.6	11.6	183.8 ± 6.87	82.6	3.74		
TCPP	0	27.3 ± 1.84	—	6.74	52.3 ± 1.34	—	2.56	158.4 ± 7.88	—	4.97	86.0 ± 4.21	85.3 ± 5.00
	50	70.0 ± 3.05	85.3	4.36	94.8 ± 9.13	84.9	9.64	202.5 ± 9.67	88.2	4.78		
	200	190.7 ± 8.60	81.7	4.51	212.4 ± 22.2	80.0	10.5	353.9 ± 46.0	97.7	13.0		
TDCP	0	nd	—	—	nd	—	—	104.8 ± 13.7	—	13.1	89.1 ± 4.47	92.5 ± 6.43
	500	475.8 ± 52.7	95.2	11.1	466.6 ± 42.8	93.3	9.17	539.7 ± 39.7	87.0	7.35		
	2000	1837.7 ± 248.5	91.9	13.5	1702.3 ± 190.6	85.1	11.2	1930.0 ± 186.8	91.3	9.68		
TPhP	0	< LOQ	—	—	< LOQ	—	—	15.9 ± 1.41	—	8.85	95.1 ± 2.57	90.2 ± 5.28
	50	44.1 ± 3.04	88.2	6.88	42.8 ± 1.09	85.6	2.56	63.1 ± 6.59	94.4	10.4		
	200	184.1 ± 16.1	92.0	8.77	183.4 ± 12.0	91.7	6.53	193.9 ± 14.6	89.0	7.54		
TiBP	0	22.5 ± 1.64	—	7.28	46.5 ± 2.41	—	5.19	456.3 ± 40.5	—	8.87	87.0 ± 1.39	91.2 ± 5.15
	50	65.6 ± 4.54	86.2	6.92	86.1 ± 5.74	79.3	6.67	497.6 ± 42.0	82.5	8.45		
	200	200.1 ± 10.1	88.8	5.05	219.8 ± 21.9	86.6	9.94	630.6 ± 13.1	87.1	2.08		
TnBP	0	13.6 ± 1.06	—	7.82	28.4 ± 2.47	—	8.68	389.4 ± 43.1	—	11.1	89.6 ± 3.56	94.6 ± 5.69
	50	56.5 ± 6.88	85.9	12.2	74.9 ± 7.61	93.0	10.2	435.9 ± 19.4	92.9	4.45		
	200	185.8 ± 23.9	86.1	12.9	205.8 ± 13.7	88.7	6.68	564.6 ± 57.0	87.6	10.1		
TBEP	0	< LOQ	—	—	11.3 ± 0.55	—	4.88	89.6 ± 8.70	—	9.71	91.7 ± 1.90	97.9 ± 5.16
	50	46.6 ± 5.87	93.2	12.6	51.7 ± 2.39	80.7	4.62	132.4 ± 17.7	85.6	13.4		
	200	170.5 ± 24.8	85.3	14.5	184.1 ± 18.9	86.4	10.3	245.9 ± 14.6	78.1	5.96		
TCrP	0	nd	—	—	nd	—	—	14.5 ± 1.63	—	11.2	87.1 ± 6.01	93.0 ± 5.20
	50	45.4 ± 3.99	90.7	8.80	39.2 ± 5.06	78.3	12.9	55.6 ± 5.98	82.2	10.8		
	200	167.1 ± 10.6	83.6	6.34	175.4 ± 9.83	87.7	5.61	196.3 ± 8.50	90.9	4.33		
EHDPP	0	nd	—	—	nd	—	—	21.3 ± 0.98	—	4.60	93.3 ± 6.53	89.0 ± 4.21
	50	39.8 ± 3.34	79.7	8.37	38.4 ± 3.50	76.8	9.13	66.3 ± 7.27	90.1	11.0		
	200	170.3 ± 10.3	85.1	6.06	187.9 ± 7.42	93.9	3.95	209.9 ± 16.7	94.3	7.94		
TEHP	0	nd	—	—	nd	—	—	10.9 ± 1.13	—	10.4	88.5 ± 3.93	97.7 ± 3.72
	50	42.5 ± 1.60	85.0	3.76	38.4 ± 3.40	76.8	8.85	53.0 ± 5.00	84.2	9.43		
	200	157.5 ± 14.9	78.7	9.46	174.9 ± 8.05	87.4	4.60	187.0 ± 15.3	88.1	8.16		

In tap water, TEP, TCPP, tri-iso-butyl phosphate (TiBP), and TnBP were detected, and their concentrations were 24.2, 27.3, 22.5, and 13.6 ng/L, respectively. TCEP, TPrP, triphenyl phosphate (TPhP), and tri-butoxyethyl phosphate (TBEP) were found in tap water, but their levels were lower than the LOQ. Other OPEs were not found in the tested samples. In river water, the results were similar to those of tap water, except that TPPO was detected and TBEP was quantified. The concentrations of detected OPEs ranged from 11.3 ng/L to 52.3 ng/L. In short, some OPEs were detected at low concentrations in tap water and river water. For the effluent of sewage treatment plant, 13 OPEs were detected, and their concentrations ranged from 10.9 ng/L to 456.3 ng/L. Among them, the concentrations of TBP (TiBP and TnBP) and chlorinated alkyl OPEs (TCEP, TCPP, and TDCP) were the highest. These findings indicated that the current technology has a limited effect to remove OPEs. The sewage treatment plant should be improved.

### Comparison with other methods

At present, various pretreatment methods have been used to extract OPEs in aqueous samples, and their main parameters are shown in Table 3. LLE has good recoveries and relatively low LOQs but requires considerable amounts of aqueous samples and consumes more organic solvents compared with other methods^24^. SPE has low LOD and LOQ and good recoveries for most OPEs but also requires more aqueous samples than other methods^38^. SPME does not consume organic solvent, requires small amounts of aqueous samples, and has good recoveries and relatively low LOQs. However, it is time-consuming. The commercialized SPME fiber can be only extracted by approximately 50 times and is very fragile. A homemade SPME fiber has longer service life and better stability but requires a cumbersome preparation process^28,39^. DLLME requires small amounts of aqueous samples and organic solvents and has relatively high recoveries and low LOD and LOQ. However, the traditional DLLME uses highly toxic chlorinated solvents, such as trichloroethane, as the extractant^40^. DLLME-SFO uses less toxic organic solvents, such as undecanol, as the extractant but requires centrifugation to separate the extractant from aqueous samples, making it unsuitable in field applications^31^. Compared with the above methods, SD-DLLME-SFO requires small amounts of aqueous samples and has shorter extraction time; environment friendly extraction solvent; and reasonable recoveries, LOD, LOQ, and RSDs. Therefore, SD-DLLME-SFO is a suitable method to detect OPEs in aqueous samples.

**Table 3:** Comparison of the proposed method with other methods for determination of OPEs in aqueous samples.

Methods	Field applicability	Sample volume (mL)	Extraction time (min)	Extraction solvent	Solvent volume (mL)	Separation method	LOD (ng/L)	LOQ (ng/L)	Recoveries (%)	RSD (%)	OPE numbers	Ref
LLE-LC-MS/MS	Yes	800	—	Dichloromethane	35	—	—	2.6–7.9	63–94	1.9–12	9	^24^
SPE-GC-MS	No	1000	—	Ethyl acetate	4	—	0.006–0.85	0.015–2.0	31.2–81.4	2.9–9.9	8	^38^
DI-SPME-GC-NPD	Yes	22	40	—	—	—	—	10–25	26.7–119.2	<10	9	^28^
HS-SPME-GC-NPD	Yes	10	40	—	—	—	1.4–135.6	4.7–452.0	76.4–112.4	<9.8	9	^39^
DLLME-GC-NPD	No	10	1	1-Trichloroethane	0.02	Centrifugation	—	10–80	—	<10	10	^40^
DLLME-SFO-LC-MS/MS	No	8	2	1-Undecanol	0.4	Centrifugation	20–70	-	48.7–113	3.2–12.3	8	^31^
SD-DLLME-SFO-LC-MS/MS	Yes	10	3	1-Undecanol	0.075	Demulsification	0.16–20	0.55–66.7	68.2–97.7	<15	13	This study

## Experimental

### Reagents and materials

The name, abbreviation, and CAS number of the 13 OPEs are as follows: triethyl phosphate (TEP, 78–40–0), tripropyl phosphate (TPrP, 513-08-6), tri-iso-butyl phosphate (TiBP, 126-71-6), tributyl phosphate (TnBP, 126-73-8), tris-(2-chloroethyl)phosphate (TCEP, 115-96-8), tris-(1-chloro-2-propyl)phosphate (TCPP, 13674-84-5), tris[2-chloro-1-(chloromethyl) ethyl]phosphate (TDCP, 13674-87-8), tri-butoxyethyl phosphate (TBEP, 78-51-3), triphenyl phosphate (TPhP, 115-86-6), 2-ethylhexyl diphenyl phosphate (EHDPP, 1241-94-7), tri(2-ethylhexyl) phosphate (TEHP, 78-42-2), triphenylphosphine oxide (TPPO, 791-28-6), and tricresyl phosphate (TCrP, 1330-78-5). TEP, TPrP, TnBP, TCEP, TCPP, TDCP, TBEP, TPhP, EHDPP, TEHP, and TCrP were all purchased from Dr. Ehrenstorfer (Augsburg, Germany). TiBP and TPPO were purchased from Toronto Research Chemicals (Toronto, Canada). The deuterated surrogate, TnBP-*d*_27_, was obtained from C/D/N Isotopes Inc. (Pointe-Claire, QC, Canada). Acetonitrile, methanol, and acetone were purchased from Fisher Scientific (Shanghai, China). 1-Undecanol, 1-dodecanol, n-hexadecane, n-nonanoic acid, and n-octanoic acid were purchased from CNW Technologies (Düsseldorf, Germany). All organic solvents were chromatographic grade. HCl and NaOH were purchased from Sinopharm (Shanghai, China), which were reagent grade. Ultra-pure water (18.25 MΩ) was obtained from a Milli-Q Gradient system (Millipore, Bedford, USA) in our laboratory.

### SD-DLLME-SFO procedure

A 10 mL filtered water sample was poured in a glass tube; its pH was adjusted to 6.0 by adding 1 mol/L HCl. Then, 10 μL of TnBP-*d*_27_ (1 mg/L) was spiked as the surrogate into the aqueous samples. After mixing, a mixture of acetonitrile (1000 μL) and 1-undecanol (75 μL) was rapidly injected into the aqueous samples by using a syringe. Then, the samples were extracted under ambient temperature for 3 min. After extraction, 750 μL of acetone was injected as demulsifier into the aqueous samples to separate the organic solvent and aqueous samples. Then, the glass tube was transferred to an ice bath for 5 min, and the extractant was solidified and transferred to an EP tube and redissolved in 100 μL of methanol. Ten microliters of the final solution were injected into the UHPLC-MS/MS system.

### UHPLC-MS/MS analysis

A UHPLC system (Ultimate 3000, Thermo Scientific, USA) coupled with a triple quadrupole mass spectrometer (TSQ Endura, Thermo Scientific, USA) was used for the analysis of OPEs. The LC column was a Hypersil GOLD C18 column (2.1 mm × 100 mm, 1.9 μm). The column temperature was 40 °C. Mobile phase A was an aqueous solution of 0.1% formic acid, and phase B was methanol. The flow rate was 0.3 mL/min. Gradient elution was set as follows: 0 min, 40% B; 5 min, 40% B; 14.5 min, 90% B; 20.5 min, 90% B; 20.6 min, 40% B; 23.5 min, 40% B. Electrospray ionization was selected and run in positive ion mode. The peak width resolution was 0.7 m/z, spray voltage was 3500 V, sheath gas pressure was 30 arbitrary unit (Arb), auxiliary gas pressure was 7 Arb, ion transfer tube temperature was 350 °C, vaporizer temperature was 300 °C, and collision-induced dissociation gas pressure was 2 mTorr. The selective reaction monitoring transitions are listed in Table 4.

**Table 4:** The UHPLC-ESI^+^-MS/MS detection parameters.

Compound	Retention Time (min)	Transitions	Collision energy (V)
TEP	2.95	183.275 → 99.000^a^	17.99
		183.275 → 127.000	10.25
		183.275 → 155.000	10.25
TCEP	4.86	284.912 → 222.835^a^	12.28
		284.912 → 99.000	22.84
		284.912 → 160.889	15.21
TPPO	8.52	279.005 → 200.946^a^	25.78
		279.005 → 172.929	33.11
		279.005 → 171.018	37.46
TPrP	8.90	225.355 → 99.000^a^	17.74
		225.355 → 141.000	10.25
		225.355 → 183.000	10.25
TCPP	8.97	326.950 → 99.000^a^	22.13
		326.950 → 174.889	11.72
		326.950 → 250.8.5	10.25
TDCP	10.29	432.862 → 99.002^a^	25.67
		432.862 → 320.764	10.25
		432.862 → 322.706	10.25
TPhP	10.35	327.005 → 152.000^a^	37.2
		327.005 → 214.875	25.98
		327.005 → 250.857	26.23
TiBP	10.99	267.085 → 99.000^a^	17.53
		267.085 → 154.946	10.25
		267.085 → 211.000	10.25
TnBP-*d*_27_	11.00	294.250 → 101.986^a^	19.86
		294.250 → 230.040	10.25
		294.250 → 166.000	10.25
TnBP	11.11	267.085 → 99.000^a^	17.74
		267.085 → 154.929	10.25
		267.085 → 211.000	10.25
TBEP	11.45	399.145 → 299.000^a^	11.92
		399.145 → 198.946	14.9
		399.145 → 45.373	21.33
TCrP	12.03	369.035 → 164.986^a^	44.13
		369.035 → 165.982	29.31
		369.035 → 243.250	27.39
EHDPP	12.52	363.075 → 250.889^a^	10.25
		363.075 → 151.986	41.15
		363.075 → 214.889	31.79
TEHP	16.53	435.268 → 99.000^a^	17.03
		435.268 → 210.929	10.25
		435.268 → 323.040	10.25

^a^Transitions for quantification.

### Quantification and quality control

Procedural blanks were analyzed to determine possible contamination during extraction. The results showed that the background pollution of the proposed method was low. The main background contamination was TiBP (0.42 ± 0.05 ng/L) and TnBP (0.39 ± 0.04 ng/L), which was lower than the LOQ. TPPO, TPhP, TCPP, TDCP, and TEHP were lower than the LOD. During analysis, the background concentrations were deducted from aqueous samples. Actual aqueous samples were extracted under the most suitable conditions to determine the matrix effect. Before UHPLC-MS/MS analysis, the analytes (13 target OPEs and 1 surrogate standard) were added to the extracts. The added amount was 5 and 20 ng for each target OPE. The matrix effect was calculated using the following equation^41^:

$${\rm{Matrix}}\,\mathrm{effect}\,( \% )=\frac{{\rm{Peak}}\,{\rm{area}}\,{\rm{of}}\,{\rm{post}}-{\rm{extraction}}\,{\rm{spike}}-{\rm{Peak}}\,{\rm{area}}\,{\rm{of}}\,{\rm{real}}\,{\rm{sample}}}{{\rm{Peak}}\,{\rm{area}}\,{\rm{of}}\,{\rm{standard}}}\times 100$$

During analysis, each sample was analyzed in three replicates. Each batch of 10 samples was added with one procedural blank to monitor the potential contamination.

## Conclusion

In this study, a novel SD-DLLME-SFO pretreatment method coupled with UHPLC-MS/MS was developed for the determination of 13 OPEs in aqueous samples. The SD-DLLME-SFO process was optimized, including the type and volume of extractant, dispersant and demulsifier, extraction time, and pH of samples. Using the proposed method, the LOD and LOQ were 0.16–20.0 and 0.55–66.7 ng/L, respectively. The EFs ranged from 30 to 46. RSDs were less than 15%. The recoveries ranged from 68.2% to 97.7% in the analysis of actual aqueous samples. Effluent samples had the highest detected concentrations and types of OPEs compared with tap water and river water.

## Data Availability

The datasets generated and/or analysed during this study are available from the corresponding author on reasonable request.

## References

[1] Makinen MS (2009). Respiratory and dermal exposure to organophosphoras flame retardants and tetrabromobkphenol A at five work environments. Environ. Sci. Technol..

[2] Fan X, Kubwabo C, Rasmussen PE, Wu F (2014). Simultaneous determination of thirteen organophosphate esters in settled indoor house dust and a comparison between two sampling techniques. Sci. Total Environ..

[3] O’Brien JW (2015). Wastewater analysis of Census day samples to investigate per capita input of organophosphoms flame retardants and plasticizes into wastewater. Chemosphere.

[4] Zeng XY (2015). The occurrence and removal of organophosphate ester flame retardants/plasticizers in a municipal wastewater treatment plant in the Pearl River Delta, China. J. Environ. Sci. Heal. A.

[5] Cristale J, Katsoyiannis A, Sweetman AJ, Jones KC, Lacorte S (2013). Occurrence and risk assessment of organophosphorus and brominated flame retardants in the River Aire (UK). Environ. Pollut..

[6] Regnery J, Puttmann W (2010). Occurrence and fate of organophosphorus flame retardants and plasticize in urban and remote surface waters in Germany. Water Res..

[7] Wang RM (2015). Occurrence and spatial distribution of organophosphate ester flame retardants and plasticizers in 40 rivers draining into the Bohai Sea, north China. Environ. Pollut..

[8] Cao S (2012). Levels and distributions of organophosphate flame retardants and plasticizers in sediment from Taihu Lake, China. Environ. Toxicol. Chem..

[9] Peverly AA (2015). Chicago’s sanitary and ship canal sediment: polycyclic aromatic hydrocarbons, polychlorinated biphenyls, brominated flame retardants, and organophosphate esters. Chemosphere.

[10] Tan XX (2016). Distribution of organophosphorus flame retardants in sediments from the Pearl River Delta in South China. Sci. Total Environ..

[11] Fries E, Mihajlovic I (2011). Pollution of soils with organophosphorus flame retardants and plasticizers. J. Environ. Monitor..

[12] Luo Q (2018). Levels, distribution, and sources of organophosphate flame retardants and plasticizers in urban soils of Shenyang, China. Environ. Sci. and Pollut. Res..

[13] Mihajlovic I, Fries E (2012). Atmospheric deposition of chlorinated organophosphate flame retardants (OFR) onto soils. Atmos. Environ..

[14] Castro-Jiménez J, Berrojalbiz N, Pizarro M, Dachs J (2014). Organophosphate ester (OPE) flame retardants and plasticizers in the open Mediterranean and Black Seas atmosphere. Environ. Sci. Technol..

[15] Castro-Jiménez J (2016). Organophosphate ester flame retardants and plasticizers in the global oceanic atmosphere. Environ. Sci. Technol..

[16] Yang R, Ding J, Huang W, Xie W, Liu W (2014). Particle size-specific distributions and preliminary exposure assessments of organophosphate flame retardants in office air particulate matter. Environ. Sci. Technol..

[17] He CT (2015). Occurrence of organophosphorus name retardants in indoor dust in multiple microenvironments of southern China and implications for human exposure. Chemosphere.

[18] Brommer S, Harrad S, Van den Eede N, Covaci A (2012). Concentrations of organophosphate esters and brominated flame retardants in German indoor dust samples. J. Environ. Monitor..

[19] Salamova A, Ma Y, Venier M, Hites RA (2014). High levels of organophosphate flame retardants in the Great Lakes atmosphere. Environ. Sci. Tech. Let..

[20] Salamova A, Hermanson MH, Hites RA (2014). Organophosphate and halogenated flame retardants in atmospheric particles from a European Arctic site. Environ. Sci. Technol..

[21] Quintana JB, Rodil R, Lopez-Mahia R, Muniategui-Lorenzo S, Prada-Rodríguez D (2007). Optimization of a selective method for the determination of organophosphorous trimesters in outdoor particulate samples by pressurized liquid extraction and large-volume injection gas chromatography-positive chemical ionisation-tandem mass spectrometry. Anal. Bioanal. Chem..

[22] Van der Veen I, De Boer J (2012). Phosphorus flame retardants: properties, production, environmental occurrence, toxicity and analysis. Chemosphere.

[23] Zhang Q (2016). Thyroid hormone-disrupting activity and ecological risk assessment of phosphorus-containing flame retardants by *in vitro*, *in vivo* and in silico approaches. Environ. Pollut..

[24] Martínez-Carballo E, González-Barreiro C, Sitka A, Scharf S, Gans O (2007). Determination of selected organophosphate esters in the aquatic environment of Austria. Sci. Total Environ..

[25] Ding J, Shen X, Liu W, Covaci A, Yang F (2015). Occurrence and risk assessment of organophosphate esters in drinking water from eastern china. Sci. Total Environ..

[26] Li J (2014). Occurrence of organophosphate flame retardants in drinking water from china. Water Res..

[27] Shi Y (2016). Occurrence, distribution and seasonal variation of organophosphate flame retardants and plasticizers in urban surface water in beijing, china. Environ. Pollut..

[28] Rodríguez I (2006). Suitability of solid-phase microextraction for the determination of organophosphate flame retardants and plasticizers in water samples. J. Chromatogr. A.

[29] Tsao YC, Wang YC, Wu SF, Ding WH (2011). Microwave-assisted headspace solid-phase microextraction for the rapid determination of organophosphate esters in aqueous samples by gas chromatography-mass spectrometry. Talanta.

[30] García-López M, Rodríguez I, Cela R (2007). Microwave-assisted extraction of organophosphate flame retardants and plasticizers from indoor dust samples. J. Chromatogr. A.

[31] Luo H (2014). Dispersive liquid-liquid microextraction combined with ultrahigh performance liquid chromatography/tandem mass spectrometry for determination of organophosphate esters in aqueous samples. Sci. World J..

[32] Pang L, Yang H, Yang P, Zhang H, Zhao J (2017). Trace determination of organophosphate esters in white wine, red wine, and beer samples using dispersive liquid-liquid microextraction combined with ultra-high-performance liquid chromatography-tandem mass spectrometry. Food Chem..

[33] Wang X, Wang Y, Zou X, Cao Y (2014). Improved dispersive liquid-iquid microextraction based on the solidification of floating organic droplet method with a binary mixed solvent applied for determination of nicotine and cotinine in urine. Anal. Methods.

[34] Leong MI, Huang SD (2009). Dispersive liquid–liquid microextraction method based on solidification of floating organic drop for extraction of organochlorine pesticides in water samples. J. Chromatogr. A.

[35] Xu H, Ding ZQ, Lv L, Song DD, Feng YQ (2009). A novel dispersive liquid-liquid microextraction based on solidification of floating organic droplet method for determination of polycyclic aromatic hydrocarbons in aqueous samples. Anal. Chim. Acta.

[36] Li Y, Zhu J, Ren L, Li YX, Zou XL (2016). Application of solvent demulsification-dispersive liquid-liquid microextraction based on solidification of floating organic drop coupled with high perfomence liquid chromatography in determination of sulfonylurea herbicides in water and soil. J. Braz. Chem. Soc.

[37] Reemtsma T, Quintana JB, Rodil R, Garcı´A-López M, Rodrı´Guez I (2008). Organophosphorus flame retardants and plasticizers in water and air i. occurrence and fate. Trac-Trend Anal. Chem..

[38] Yan X (2012). Determination of Organophosphorus Flame Retardants in Surface Water by Solid Phase Extraction Coupled with Gas Chromatography-Mass Spectrometry. Chinese J. Anal. Chem..

[39] Jin T (2016). Graphene oxide based sol-gel stainless steel fiber for the headspace solid-phase microextraction of organophosphate ester flame retardants in water samples. J.Chromatogr. A.

[40] García-López M, Rodríguez I, Cela R (2007). Development of a dispersive liquid–liquid microextraction method for organophosphorus flame retardants and plasticizers determination in water samples. J. Chromatogr. A.

[41] Matuszewski BK, Constanzer ML, Chavezeng CM (2003). Strategies for the assessment of matrix effect in quantitative bioanalytical methods based on HPLC-MS/MS. Anal. Chem..

